# Demand for a Housing Offer Addressed to Senior Citizens in Poland

**DOI:** 10.3390/ijerph16224573

**Published:** 2019-11-19

**Authors:** Katarzyna Przybyła, Maria Hełdak, Izabela Kurtyka-Marcak

**Affiliations:** 1 Department of Spatial Economy, Wroclaw University of Environmental and Life Sciences, 50-375 Wrocław, Poland; 2 Institute of Economics Sciences, Wroclaw University of Environmental and Life Sciences, 50-375 Wrocław, Poland; izabela.kurtyka-marcak@upwr.edu.pl

**Keywords:** housing estate for senior citizens, housing offer addressed to senior citizens

## Abstract

The purpose of this study is to identify the willingness of pensioners to move from an apartment/house to an apartment/house located in a housing estate specifically designed for senior citizens. As such, this article forms part of the discussion of the housing needs of senior citizens, their preferences and willingness to change their place of residence. The research covers a group of post-working-age people residing in the region of Lower Silesia in south-western Poland. Various research methods were used in the study, including a written questionnaire and its statistical analysis. The research focuses on analysing both the preferences of potential buyers of apartments for seniors and seniors’ willingness to change their place of residence in order to purchase an apartment adapted to their mobility needs (free from architectural and technical barriers), taking into account the respondent’s age and housing situation. The research indicates that city residents are significantly more often willing to change their current place of residence for one adapted to the needs of persons with disabilities than residents of rural areas. In turn, those living with their husband or wife are significantly more likely to state that special offers for seniors do not need to be developed than those living alone or with other family members or in a social care home. No relationship was found between the age of a respondent and the desire to move to a new house. However, younger seniors are more open to moving to housing estates offering facilities for the elderly who require additional care.

## 1. Introduction

### 1.1. Housing Situation of Senior Citizens in Poland

Polish society is aging, which also corresponds to the European trends [1]. The ongoing aging of Polish population results from a favourable phenomenon, i.e., longer life expectancy and the deepening low fertility rate. Poland is still perceived as a demographically young country in Europe. However, since the beginning of the 1990s, the average Polish citizen has aged by over 7 years [2]. The elderly, especially those who live alone, are in need of special conditions and amenities. This has been noticed by developers who are slowly making an offer addressed to this group of customers. A special offer to purchase an apartment in a housing estate adapted to the needs of older people can attract an increasing social group each year. Residential estates can function next to typical retirement homes, which are currently more common in Poland than apartments adapted to the needs of senior citizens.

The economic possibilities presented by pensioners remain yet another problem. Despite the aforementioned increasing average income in Poland in recent decades, the elderly represent a social group characterised by significantly lower income and resources [3], and thus by a reduced ability to meet needs, sometimes living at risk of poverty. Such deficits limit mobility and the possibility of social participation [4,5].

In view of the encountered problems in the system of public services, the discussed group of people cannot always expect sufficiently effective care [6]. At the same time, it is worth emphasizing the problem of existing spatial diversity in meeting social needs which determine the living standard of the population, including senior citizens [7,8,9,10]. Therefore, changing an apartment/house should be analysed more often than purchasing a new one.

The current state housing policy, addressed to senior citizens, allows adapting an apartment to age-related mobility limitations, as long as the person has a disability certificate. Activities focused on eliminating architectural barriers in seniors’ place of residence are particularly important in the context of improving their quality of life and independence [11,12]. The elderly, uncared for by their families, have nursing homes at their disposal in Poland, as long as they can afford to live in such institutions (i.e., receive a pension covering the costs of their stay). The state has not provided any support programme addressed, for example, to developers that would encourage them to develop a preferential housing offer for seniors: no preferential loans or tax reliefs. As emphasised by Park, S. et al. [13], empirical knowledge of the correlation between subsidised senior housing and its neighbourhood is an important initial contribution to the policy-level intervention of optimizing subsidised senior housing in an effort to overcome the socio-economic disparity at the neighbourhood level. Because of insufficient financial resources, lower socioeconomic status (SES) elders in conventional homes often perceive aging in their current home as the only choice [13], even if they are unable to modify their homes and access services in their neighbourhoods. It can also be inferred that this financial barrier exerts impact on the choice of residence for seniors, despite their apartment not having been adapted to their physical needs.

### 1.2. Housing Preferences of Senior Citizens

Recent studies highlight that people prefer “aging in place”. This is defined as “remaining living in the community, with some level of independence, rather than in residential care” [14,15]. Lawler [16] approaches it as allowing seniors to remain independent, autonomous and at the same time connect with social support. There is also a widespread opinion that helping people to carry on living in their own homes is desirable for economic reasons, because it is cheaper than such options as care in a social care home (or other social housing) [17]. Research focused on determining factors with a positive impact on elderly independent living is not only carried out in Europe, but also worldwide [11,18,19]. This allows determining what kind of solutions should be used to adopt individual apartments or houses as well as entire cities to the needs of senior citizens [19]. Developing a special housing offer in the private sector is discussed in the presented article. The development of a social housing offer for seniors was not included in the research, but rather their willingness to change and the demand for a housing offer created by developers.

According to Wiles J.L et al. [20], there is a strong focus on housing and support or care in the aging-in-place research literature [21,22].

The research motive is the mobility problem of the elderly and the assessment of their willingness to change their place of residence after retirement into a private apartment adapted to the needs of senior citizens. The authors analyse whether Polish pensioners are mobile and ready to change their apartment. It seems that along with the increasing affluence of Polish society [23,24], such processes should intensify. However, the research carried out by Wiles [20] shows that many seniors expressed a strong desire to remain in their own homes, linked to a sense of independence and autonomy. Often this was as much about not wanting to be in a nursing home or institution, where it was perceived that autonomy might be lost, as about remaining in the same place.

The possibility to remain at home depends on the ability to function in late life and have access to home- and community-based services, which facilitates independence and autonomy [25]. Many older adults prefer aging in place in familiar housing and the communities to which they are attached [26,27].

The authors of the recently published studies [28] present the following opinion: ageing in place is an idea evolving in Western Europe, according to which the elderly may maintain independence and self-reliance by adapting their dwellings to the specific needs of older people [29,30]. The research conducted on seniors’ preferences shows that they want to live in their familiar environment as long as possible [31]. An analysis of the housing preferences of the elderly in big Polish cities (Warsaw, Szczecin, and Poznań) was carried out by Iwański, R; Rataj, Z.; Cieśla [28], but this does not cover the current housing situation of senior citizens.

### 1.3. The Purpose and Motives of the Conducted Research

The purpose of the study is to identify the willingness of pensioners to move from an apartment/house to an apartment/house located in a housing estate and the related preferences. The research is focused on determining the attitudes of post-working-age people to the more frequent market offers of apartments addressed to a specific group of customers. Basically, the studies show the need for facilities that can be implemented in a housing estate and the scale of demand for such an offer.

The research motive was the analysis carried out by Hełdak et al. [4] on the elimination of architectural and technical barriers in Poland, as well as the studies conducted by Kurtyka-Marcak et al. [12] covering the actual demand for construction works and changes in interior design aimed at eliminating mobility limitations of retirement-age people. As established, this social group rarely applies for the disability status, and the actual demand does not correspond to the number of people holding a disability certificate. The analysis of senior citizens’ health condition (in post-working age) was performed and the scale of their mobility limitations was identified. The actual demand for construction works and changes in interior design aimed at eliminating mobility limitations were indicated. The research was continued and covered the demand for a housing offer taking into account the needs of senior citizens.

The current housing stock frequently does not support the needs of older adults who prefer aging in place [32]. The problem of dementia resulting from aging is increasing all over the world. By 2030, the number of people suffering from dementia worldwide may exceed 82,000,000 [33]. Dementia and cognitive impairment contribute significantly to disability, health problems, and social care needs among older people. Therefore, it is very important to seek solutions that will ensure both a dignified and safe life for seniors.

## 2. Methods

The presented study is focused on identifying seniors’ preferences regarding sales offers of apartments/houses equipped with the prepared facilities for pensioners and also determining the demand for additional (special) services possible to implement at a senior housing estate.

The group of senior citizens includes people over 55 years of age (in Poland, this age is associated with the possibility of early retirement). Women over 55 and men over 60 can apply for early retirement. This, however, is related to documenting contributory and non-contributory periods as of 1 January 1999. The required contribution period for women is at least 20 years and for men at least 25 years. Moreover, this period has to cover 15 years of work in special conditions.

The following hypotheses were put forward in the article:

**Hypothesis** **1.** *The willingness to move from an apartment/house to an apartment/house located in a housing estate specifically designed for senior citizens is indicated primarily by the residents of large cities.*

**Hypothesis** **2.** *The willingness to move from an apartment/house to an apartment/house adapted to mobility needs declines with age.*

**Figure 1:**
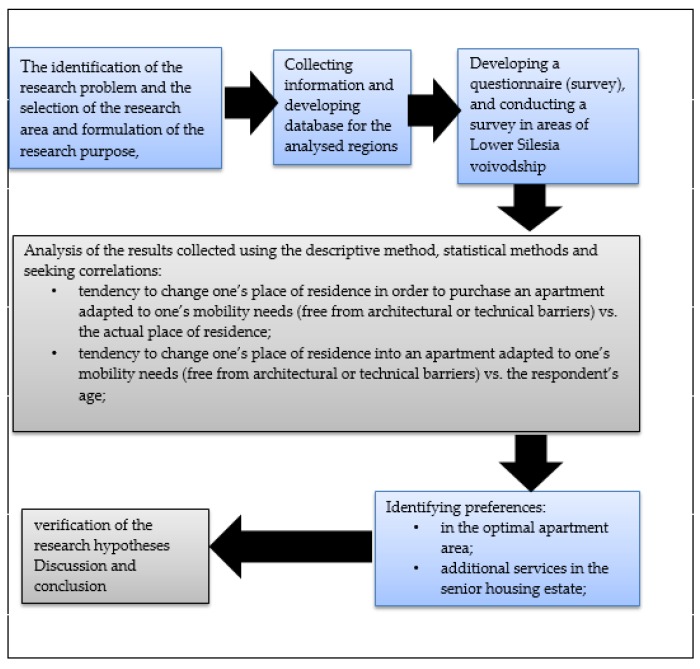
The research stages.

The method of study follows the following steps (Figure 1):

A total of 214 random respondents, within the indicated age group, participated in the survey. The authors did not establish any guidelines regarding the number of people by gender, age or place of residence. The important factor was the number of respondents. The questions addressed to the respondents are presented below (Table 1).

In order to provide answers to the presented research questions and to test the formulated hypotheses, statistical analyses were carried out using IBM SPSS Statistics 25 package version (StatSoft Polska Sp. z o.o., Kraków, Poland). 

The analysis of differences in the cross-tabulation of quality characteristics was conducted using Pearson’s chi-square test (χ^2^ test of independence) or Fisher’s exact test when the expected number was less than 5. As a post hoc analysis checking the nature of differences occurring between groups, the analyses were carried out using the method by Baesley and Schumacker [34]. The maximum permissible error and type α = 0.05 was adopted for all analyses, whereas *p* ≤ 0.05 was considered statistically significant.

## 3. The Demographic Characteristics of the Research Area

The research covered the area of Lower Silesia voivodship, located in southwestern Poland. The city of Wrocław is the capital of Lower Silesia, which is also an important administrative, academic and service centre in the country. The voivodship is ranked as fifth in Poland in terms of population. Currently, approx. 2,900,000 inhabitants (Statistics Poland) reside in Lower Silesia [35]. In 2017, 6,359,000 post-working-age people lived in Lower Silesia voivodship. At present, people aged 65 and older make up 17.7% (the respective number in 1990 was only 9.2%), whereas in cities, this share is 19.4% (8.5% in 1990), and in villages, 14.0% (11.0% in 1990) (Statistics Poland, yearbook). The forecasts prepared by Statistics Poland indicate that the discussed changes will deepen, amounting to 12 children under the age of 14 and 34 people aged 65 and over per 100 inhabitants of Lower Silesia voivodship in 2050 [35,36].

The aging process remains one of the major challenges and demographic problems faced by the voivodship [36]. The economic burden rate specifying the number of pre-working and post-working-age people per 100 working age population was 61 in 2016 (34 post-working age and 27 pre-working-age). The forecasts provided by Statistics Poland show that this indicator will continue to increase, reaching the level of 73 in 2030 and 106 in 2050, i.e., in 2050, as many as 106 post-working-age people will fall per 100 working age population (of which, 77 post-working age and 29 pre-working-age people will fall per every 100 population, representing the potential labour force resources) [36].

## 4. The Willingness to Move from an Apartment/House to an Apartment/House Adapted to the Mobility Needs Depending on the Place of Residence and Age of the Respondents

**Figure 2:**
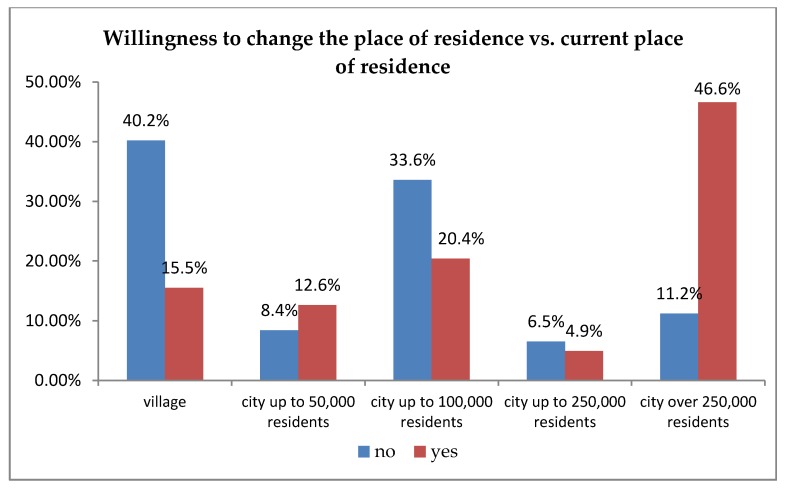
Willingness to change the place of residence in order to purchase (acquire) an apartment adapted to one’s mobility needs, free from architectural and technical barriers depending on the current place of residence.

In the conducted research, the primary focus was on the analysis of the respondents’ willingness move from an apartment/house to an apartment/house that is adapted to the needs of senior citizens, depending on the current place of residence. For this reason, the analysis using Pearson’s chi-square test of independence was carried out to verify whether the analysed respondents were willing to change their place of residence and purchase an apartment adapted to their mobility needs depending on where they currently reside. The analysis showed significant differences between the compared groups (χ^2^ (4) = 38.90; *p* < 0.001; *v* = 0.43). Figure 2 illustrates the percentage comparison of the answers provided by the respondents.

To check the nature of the occurring differences, an additional post hoc analysis was carried out taking into account the method by Baesley and Schumacker [34]. The conducted analysis showed that people living in villages and cities of up to 100000 residents are less willing to change their place of residence. People living in cities of over 250000 residents are more likely to change their place of residence. It may result from stronger social ties connecting residents in smaller territorial units and also, to some extent, from the greater wealth of larger city residents [37].

**Figure 3:**
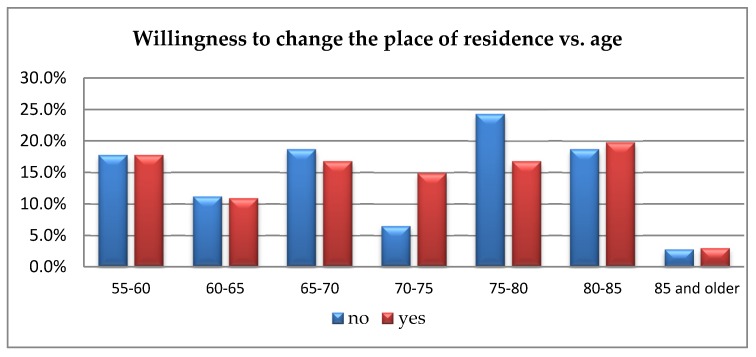
Willingness to change the place of residence in order to purchase an apartment adapted to one’s mobility needs, free from architectural and technical barriers depending on residents’ age.

The studies analogical to the aforementioned ones were conducted to determine the willingness to change the place of residence depending on age. Due to the fact that in 14.3% cells, the expected number was less than 5, the analysis was carried out using Fisher’s exact test. The analysis showed no significant differences regarding the willingness to change the place of residence depending on the respondents’ age (*p* = 0.550). Therefore, no correlation occurs between age and the willingness to move. Figure 3 illustrates the responses provided by the respondents.

The presented results confirm a considerable readiness to change the place of residence, which oscillates around the level of approx. 15%. The absence of decisive disproportions between “no” and “yes” answers to the question about the willingness move from an apartment/house to an apartment/house adapted for the elderly was observed.

The research conducted in three Polish cities (Warsaw, Szczecin, and Poznań) [28] showed that when asked about their readiness to change their current apartment, the respondents provided the following answers: nearly 70% of those living in Poznań marked “definitely not’”, while for Warsaw and Szczecin, this answer was selected by approximately half of the respondents. A quarter of the respondents from Szczecin would be willing to change their place of residence, but only if it was in the same estate/in the same neighbourhood; in Warsaw, the respective share was 16.3%, whereas in Poznań, it was 8%.

## 5. Developing a Special Sales Offer of Apartments/Houses for Senior Citizens vs. the Respondent’s Family Situation, Place of Residence, and Age

### 5.1. Family Situation vs. Opinion about Establishing Senior Housing Estates

The respondents’ opinions related to developing special sales offers of apartments/houses for seniors depending on their family situation, place of residence and age constituted the next analysed research area. The analyses were performed using Pearson’s chi-square test of independence or Fisher’s exact test also using the post-hoc procedure by Baesley and Schumacker [34]. The obtained results are discussed below.

The analysis using the chi-square test of independence showed significant differences between the responses provided by the respondents depending on their family situation χ^2^ (18) = 39.87; *p* = 0.002; *v* = 0.25. Additional post hoc analyses applying the method by Baesley and Schumacker [34] were carried out to check the nature of differences.

It is noticeable that in the majority of the studied groups (five out of seven), the most common response regarding the development of special housing offers for seniors is “yes”. If the answer “I have no opinion” is rejected, another group is included in this set (people living with children, “I have no opinion” (35.5%), “yes” (29%), to be followed by “no” (22.6%)).

Additionally, it was recorded that in the case of three of these groups (people living alone, with a husband/wife, in a nursing home), at least 50% of the respondents from a given group provided such an answer.

The results for the group of people living with a husband/wife are interesting. In total, 51% of the respondents from this group are in favour of developing a special offer, 26% of the respondents answered “maybe” and at the same time no one answered “no”. The people living in care and treatment facilities represented even stronger supporters for developing the offer. Almost 78% of them were in favour of such solution, the rest of the group did not have any opinion regarding this issue.

In the group of those responding positively to developing special housing offers for senior citizens, there are significantly fewer people living in a nursing home than the ones living in care and treatment institutions, alone, or with a spouse and other family members.

**Figure 4:**
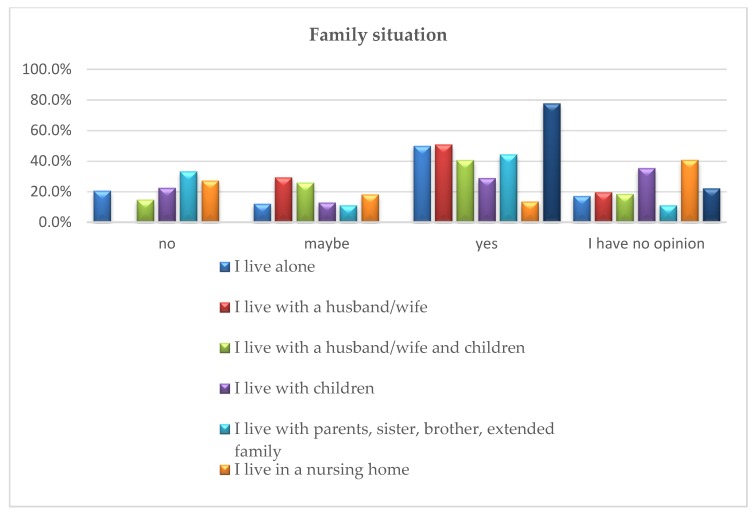
Family situation of the respondents vs. opinion about developing special sales offers of apartments/houses for senior citizens.

The answer “no” did not prevail in any of the analysed groups. The highest percentage of negative responses were provided by those living with their parents, sister, brother, extended family (33.3%), and also in the group living in a nursing home (27.3%). At the same time, those living in nursing homes answered “yes” the least often out of all the studied groups (13.6%) and most frequently had no opinion (Figure 4).

### 5.2. Current Place of Residence by the Type of Building vs. Developing a Housing Offer for Senior Citizens

The analysis carried out using the chi-square test of independence did not show significant differences in the provided opinions depending on the type of building being the respondents’ place of residence (detached house, terraced house, apartment in a multi-family building, nursing home, care and treatment facility); χ^2^ (12) = 19.83; *p* = 0.070.

**Figure 5:**
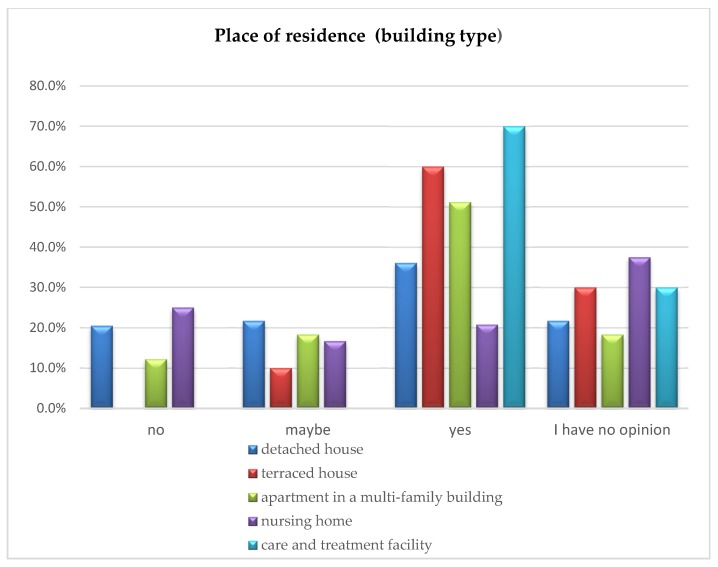
Building type of the respondents’ current place of residence vs. opinion on developing special sales offers of apartments/houses for senior citizens.

At the same time, it should be noted that only in the group of nursing home residents the percentage of people responding “no” was higher than those answering “yes”. The remaining groups provided the majority of “yes” responses. The percentage comparison of the respondents’ answers is illustrated in Figure 5.

### 5.3. The Respondents’ Age vs. Opinion on Developing a Housing Offer for Senior Citizens

**Figure 6:**
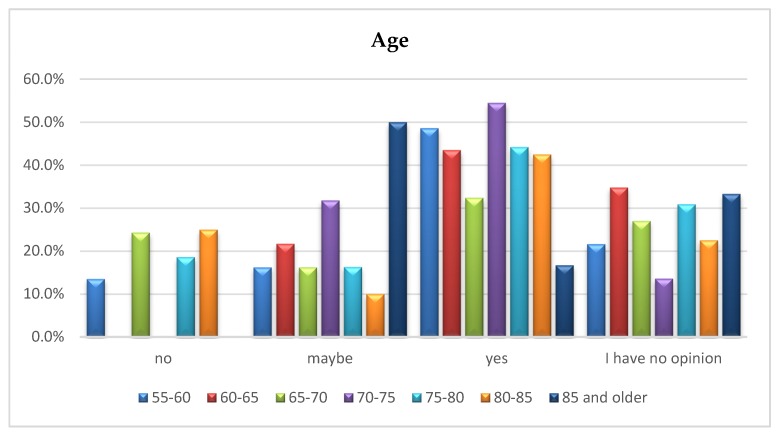
Age of the respondents vs. opinion on developing special sales offers of apartments/houses for senior citizens.

For the respondents’ opinions regarding the need to develop special housing offers for seniors depending on their age, the chi-square test of independence did not show significant differences χ^2^ (18) = 25.68; *p* = 0.107. In all age groups, except for people aged 85 and older, the opinion “yes” prevails. This exception is most likely due to the fact that the oldest people no longer see the possibility or need to change their place of residence. Figure 6 shows the respondents’ answers depending on age.

## 6. Preferred Square Footage of an Apartment/House and the Facilities It Provides

The analyses carried out by Kurtyka-Marcak et al. [12] and covering a similar group of respondents in terms of the demand for services in the immediate vicinity revealed that all the respondents, regardless of age and place of residence, indicated the need for a health centre in their close surroundings. The same was true for a grocery store (95% on average in each age group), a public transport stop (approx. 70% of the respondents) and a church (approx. 70% of the respondents).

**Figure 7:**
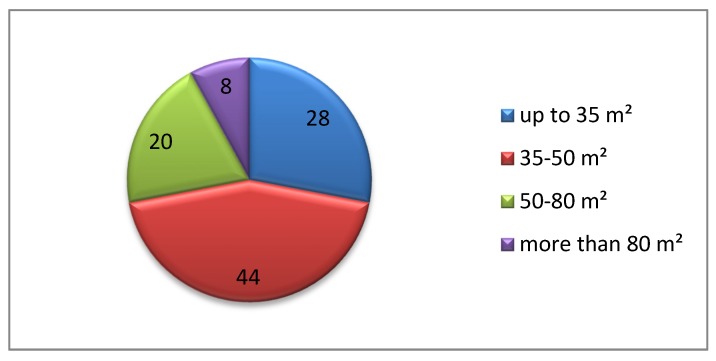
Apartment square footage at a senior housing estate preferred by the respondents.

Another analysed problem was the study of preferences regarding the apartment square footage in a senior housing estate and also in terms of special services. The analysis revealed that if the respondents decided to change or purchase an apartment, they would mostly choose the square footage of up to 50 m^2^, with the largest interest in the footage ranging from 35 to 50 m^2^ (44% of the respondents). Every fourth respondent prefers the smallest apartment of up to 35 m^2^, whereas every fifth from 50 to 80 m^2^ (Figure 7). Only 8% of the population indicated the largest square footage, i.e., more than 80 m^2^.

**Figure 8:**
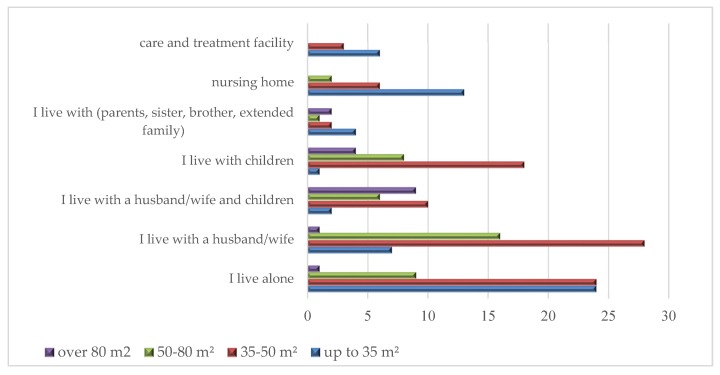
Square footage of an apartment at a senior housing estate by family situation preferred by the respondents (number of indications).

People living alone preferred an apartment square footage of up to 50 m^2^ (the total of 48 indications). Spouses without children showed the greatest interest in the footage ranging from 35 to 50 m^2^ (28 indications) and also from 50 to 80 m^2^ (16 indications). Those living with a spouse and children or living with children would choose larger apartments. Seniors staying in nursing homes and also in care and treatment facilities were predominantly interested in small apartments (Figure 8).

Housing estates addressed to older people should feature additional services improving the quality of life their residents. The survey covered additional facilities to be provided at a senior housing estate. Among the indicated ones, the following were listed: 24 h medical care (on call duty at the estate), 24 h medical care on call (button in the apartment), live-in caregiver (assistant), guarded estate, house cleaning, canteen and other facilities identified by the respondents.

Assistive technology (e.g., alarms, telecare) may also have the potential to assist in enabling older people and/or those with dementia to remain in their own homes [38,39]. Physical modifications to the original structure and design of dwellings, especially in relation to improving the accessibility of home environments, have also been shown to be key elements in facilitating aging in place [40].

**Figure 9:**
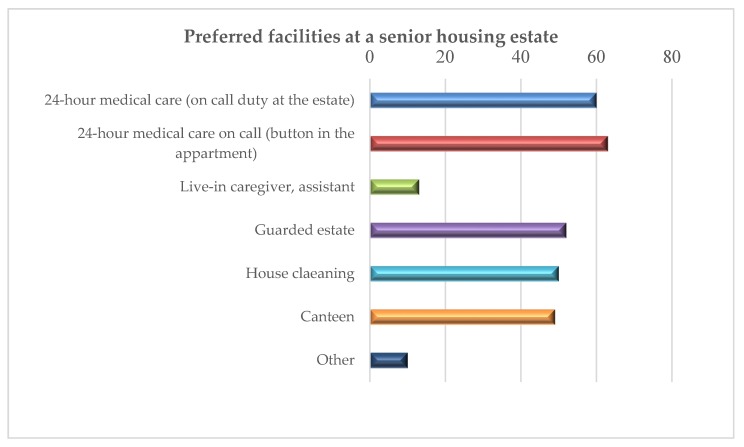
Facilities at a senior housing estate preferred by the respondents (%).

Based on the opinions presented by seniors, it was established that the most popular facilities were as follows: 24 h on-call medical care (button in the apartment) and 24 h medical care (on-call duty), i.e., 63% and 60% of the population, respectively. For half of the respondents, safety is very important because they chose an apartment in a guarded estate. The same number of respondents indicated the possibility of using a canteen and house cleaning (Figure 9). Among “other” preferred facilities, the following were listed: a restaurant (six indications), a snack bar specializing in dairy products, a park (two indications each) and a swimming pool (one indication).

**Figure 10:**
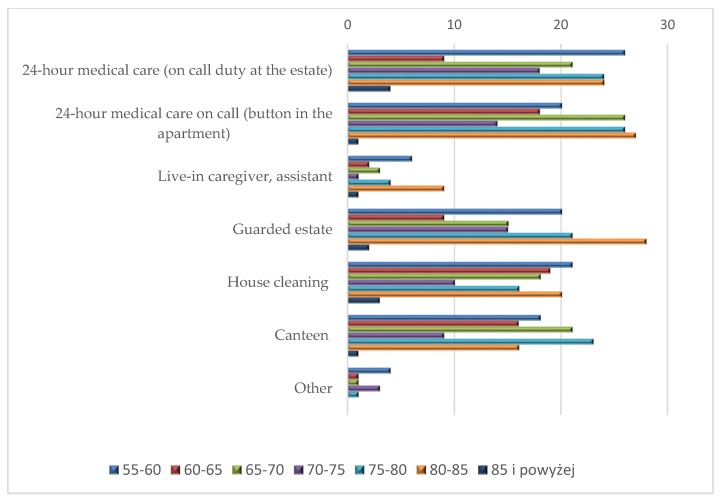
Facilities at a senior housing estate preferred by the respondents by age (number of indications).

**Figure 11:**
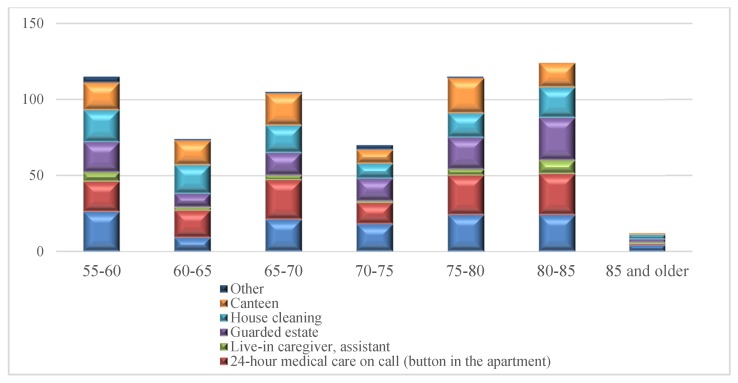
Facilities at a senior housing estate preferred by the respondents by age (number of indications).

The analysis of facilities preferred by respondents depending on age was also performed (Figure 10 and Figure 11).

The respondents aged 80–85 indicated the greatest demand for various facilities (124 indications). The majority of needs referred to a guarded housing estate (28 indications), 24 h on-call medical care (button in the apartment) (27 indications), 24 h medical care (on-call duty at the estate) (24 indications) and house cleaning (20 indications). The age groups 55–60 and 75–80 presented the same number of indications (115) and very similar preferences (Figure 9). Their most frequent choice was 24 h medical care (on call duty) (26 and 24 indications respectively), 24 h medical care on call (button in the apartment) (20 and 26 indications), guarded housing estate (20 and 21 indications), house cleaning (21 and 16 indications) and a canteen (18 and 23 indications). The importance of the aforementioned facilities for senior citizens is confirmed by the data collected within the framework of the study conducted in Poland in 2014, in line with Eurostat guidelines as part of the European Health Interview Survey (EHIS) [41]. The studies have shown that over 2,000,000 people aged 65 and older (34% of the total population) had limitations in performing basic daily activities—of which, 200,000 people had mild limitations, 1,311,000 had moderate limitations, and 504,000 people had severe limitations. The possibility of everyday independent functioning declines with age. In the age group 65–69, every fifth respondent had problems; among seventy-year-olds, almost every third respondent had problems; and in the oldest age group (80 or older), more than every second person had problems (58%). Most frequently, ailing people or those in poor health had problems in their daily functioning, which was more often signalled by older women than men. The limitations they suffer do not allow them to do heavy housework; over 57% of the respondents aged 65 and older indicated difficulties in performing such activities. Doing everyday shopping was a problem for every third elderly person, to be followed by difficulties in performing less demanding housework or taking care of financial matters and everyday administrative issues. The scale of health limitations in running a household is confirmed by the following numbers. It is estimated that 1,100,000 seniors found it difficult to prepare meals, over 810,000 had problems using their landline phone, 2,100,000 people doing daily shopping, and over 800,000 preparing and taking medicines. The scale of needs regarding this basic aspect of human life can be demonstrated by the fact that almost 45% of seniors have problems with taking basic care of themselves and have to cope on their own because they are not provided with any assistance [42].

The least preferred facility was an apartment with a live-in caregiver or an assistant. This need was indicated only by 26 respondents, mainly aged 80–85 (nine indications) and the youngest ones aged 55–60 (six indications). Small interest in this form of assistance is most likely related to the low popularity of this type of care for the elderly or the disabled in Poland, as compared to Western European countries such as Germany or the United Kingdom. Older people have a lower sense of security and are afraid to live with a stranger under one roof.

## 7. Discussion

As indicated by the demographic forecasts for the region of Lower Silesia, but also for Poland on the whole, the population age structure will change radically in the near future. This also refers to the group of senior citizens. The number of people at advanced age, i.e., 85 and over, will increase, which is associated with the aging of the birth cohorts recorded during the demographic summit after World War II [28]. Developed societies will be increasingly burdened with providing social care for the aging and growing part of the population. A much better solution seems to be the support provided in the place of residence using the care of an assistant, on-call medical service and help in everyday activities. Such services can be offered by implementing a special housing offer. Another solution is the construction of housing estates offering facilities targeted at people with additional limitations resulting from their age. The state policy should aim at supporting economic initiatives on the real estate market through preferential loans or tax reliefs.

The conducted research is crucial from the perspective of making decisions by the authorities in the area of programmes supporting the development of a new housing offer for senior citizens. In addition, they suggest areas in which such a housing offer could be created.

Hypothesis 1 was confirmed in the course of the conducted research: The willingness to move from an apartment/house to an apartment/house adapted to the mobility needs of seniors is indicated primarily by the residents of large cities. It was shown that individual groups of respondents differ significantly in this respect and that people living in villages and cities of up to 100,000 residents are less likely to change their place of residence. In turn, seniors living in cities populated by over 250,000 residents are significantly more likely to change their place of residence. This seems to result from stronger social ties connecting the inhabitants of smaller territorial units but also, to a certain extent, from the greater wealth of larger city residents.

Hypothesis 2: “The willingness to move from an apartment/house to an apartment/house adapted to mobility needs declines with age” was not confirmed. In the majority of surveyed age groups, approximately the same percentages of people indicated willingness and unwillingness to change their place of residence. Interestingly, it was also observed that in all age groups, except for people aged 85 and older, the “yes” opinion dominates regarding the development of a special housing offer for seniors. Thus, people declare that the offer addressed to the elderly should be developed. However, at the same time, the majority of them are not necessarily willing to move.

## 8. Conclusions

Based on the conducted research, the following conclusions were formulated:

1. Residents of large cities are more likely to change their place of residence in order to purchase an apartment adapted to their mobility needs (free from architectural or technical barriers).
2. The research has not shown that as people age, their willingness to change the place of residence into an apartment adapted to their mobility needs declines, and hence the housing offer can be addressed to seniors of all ages.
3. The conducted research allowed determining preferences regarding the square footage of apartments at senior housing estates and preferences for special services. Apartments ranging in size from 35 to 50 m^2^ were the most popular. Thus, such apartments should be offered predominantly at senior housing estates. The apartments up to 35 m^2^ ranked as second.
4. In the opinion of the elderly, senior housing estates should be guarded, 24 h medical care should be provided as well as a house cleaning service and a canteen. These special facilities are needed because of the reduced ability to perform various daily activities and worsening health condition.
5. The aforementioned suggestions should be taken into account while planning and constructing housing estates addressed to senior citizens by developer companies.

## Figures and Tables

**Table 1:** Questions asked in the survey.

Research on the Demand and Preferences for Purchasing Apartments Addressed to Senior Citizens
1. Do you hold a disability degree certificate?
yes
no
2. If so, the certificate states:
severe degree of disability
moderate degree of disability
mild degree of disability
3. Do you think that a special sales offer of apartments/houses for senior citizens should be developed?
yes
no
maybe
I have no opinion
4. Would you be willing to change your apartment/house into an apartment/house adapted to the needs resulting from mobility limitations?
yes
no
maybe
5. Should the apartments adapted for seniors:
be established as entire housing estates?
be established as a certain share of apartments (a part) in a housing estate, if so what share (%)?
6. Would you be willing to change your place of residence and purchase an apartment adapted to your mobility needs (free from architectural or technical barriers)?
yes
no
7. Have you ever heard of a housing offer addressed to senior citizens:
yes
no
8. If you decided to change/purchase an apartment, what square footage would you be interested in?
up to 35 m^2^
35–50 m^2^
50–80 m^2^
more than 80 m^2^
9. What public services should be available in the immediate vicinity of the senior housing estate?
Health care centre
A community centre providing activities for senior citizens
Kindergarten
Public transport stop
Taxi rank
Grocery store
Restaurant
Church
Other, what kind…
10. What additional facilities should be available at the senior housing estate?
24 h medical care (on call duty at the estate)
24 h medical care on call (button in the apartment)
Live-in caregiver, assistant
Guarded housing estate
House cleaning
Canteen
Other, what kind …
11. Respondent’s place of residence:
Detached house
Terraced house
Apartment in a multi-family building (apartment block)
Nursing home
Other, what kind…
12. Respondent’s family situation:
I live alone
I live with a husband/wife
I live with a husband/wife and children
I live with children
I live with (parents, sister, brother, extended family) * delete as appropriate
Nursing home
Other, what kind …
13. Age range:
55–60
60–65
65–70
75–80
80–85
85 and older
14. Place of residence:
Village
City up to 50,000 residents
City up to 100,000 residents
City up to 250,000 residents
City over 250,000 residents
Gender:
female
male

Source: authors’ compilation.
